# Supplementary material for: Expression Profiling of Extracellular Matrix Genes Reveals Global and Entity-Specific Characteristics in Adenoid Cystic, Mucoepidermoid and Salivary Duct Carcinomas
Source: Cancers (Basel). 2020 Aug 31;12(9):2466. doi: 10.3390/cancers12092466 (PMC7564650; doi:10.3390/cancers12092466)
Supplement: Supplementary file 1 [file cancers-12-02466-s001.zip › cancers-880335 supplementary final.docx]

**Supplementary Materials:**

Expression Profiling of Extracellular Matrix Genes Reveals Global and Entity-Specific Characteristics in Adenoid Cystic, Mucoepidermoid and Salivary Duct Carcinomas

**Figure S1.** Principal component analysis labelled according to sample age and based on log_2_-fold values from the RNA expression analysis. A two-dimensional biplot with PC1 on the x axis and PC2 on the y axis. Individual samples are shown as points, colored according to their sample date.

**Figure S2.** Principal component analysis labelled according to tumour type and based on log_2_-fold values from the RNA expression analysis. A two-dimensional biplot with PC1 on the x axis and PC2 on the y axis. Single genes are depicted as vectors, illustrating their weight on the PCs. Individual samples are shown as points, colored according to their respective histological group. AdCy: Adenoid cystic carcinoma; MuEp: Mucoepidermoid carcinoma; SaDu: Salivary duct carcinoma.

**Figure S3.** Boxplot depicting entity-wise RNA expression of all analysed laminins as log_2_-fold values. Individual cases are displayed as points. Significant differences for comparisons between two individual histological groups are given as asterisks above the boxplots (adjusted *p* values; *: *p* < 0.05; ns: not significant). Significance levels for comparisons between tumor and normal tissue are given as number symbols below the boxplots (adjusted *p* values; #: *p* < 0.05; ##: *p* < 0.01; ###: *p* < 0.001; ns: not significant). AdCy: Adenoid cystic carcinoma; MuEp: Mucoepidermoid carcinoma; SaDu: Salivary duct carcinoma.

**Figure S4.** Boxplot depicting entity-wise RNA expression of all other analysed glycoproteins as log_2_-fold values. Individual cases are displayed as points. Significant differences for comparisons between two individual histological groups are given as asterisks above the boxplots (adjusted p values; *: *p* < 0.05; ns: not significant). Significance levels for comparisons between tumor and normal tissue are given as number symbols below the boxplots (adjusted p values; #: *p* < 0.05; ##: *p* < 0.01; ###: *p* < 0.001; ns: not significant). AdCy: Adenoid cystic carcinoma; MuEp: Mucoepidermoid carcinoma; SaDu: Salivary duct carcinoma.


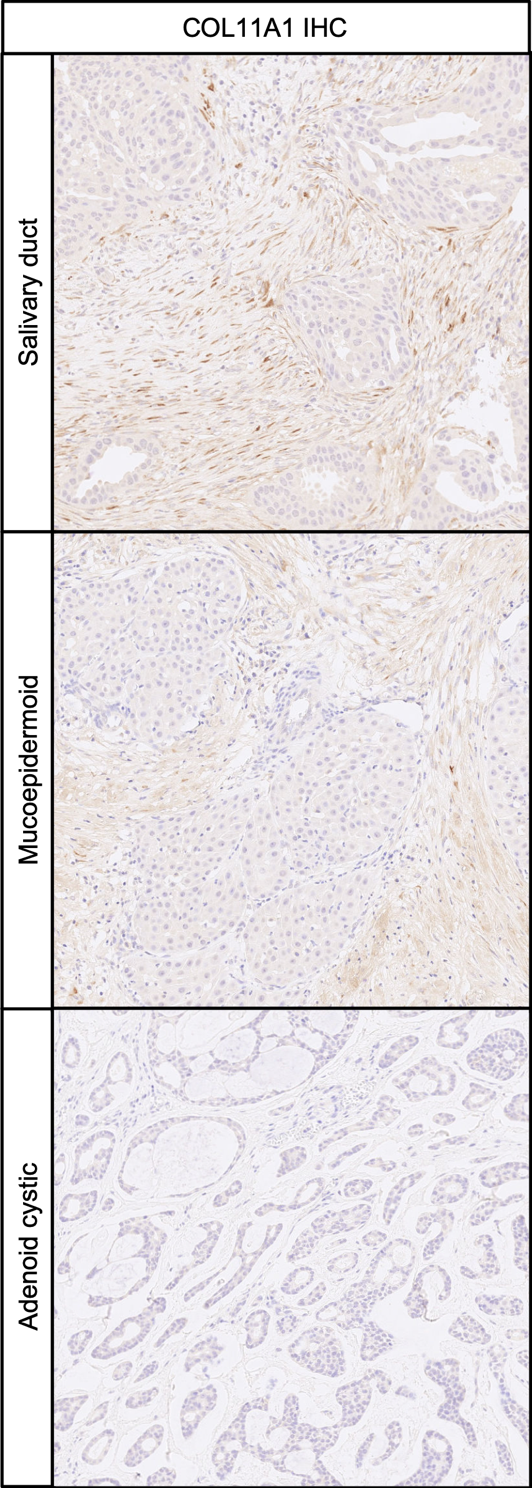


**Figure S5.** In situ staining of COL11A1 protein. Salivary duct (top), mucoepidermoid (middle) and adenoid cystic carcinoma (bottom); Magnification: 100 x.

**Table S1.** Overview of the 10 most differentially expressed genes per histological group. Adjusted *p* values <0.05 as well as log_2_-fold values above 2 are highlighted in bold type. No genes were downregulated below a log_2_-fold value of <–2. SaDu: Salivary duct carcinoma; MuEp: Mucoepidermoid carcinoma; AdCy: Adenoid cystic carcinoma**.**

|  | **Gene** | ***p* Adj** | **Log_2_fold** |
| --- | --- | --- | --- |
| SaDu |  |  |  |
|  | COL1A1 | **0.00029** | **6.16** |
|  | COMP | **0.00040** | **5.80** |
|  | SPP1 | **0.00040** | **5.25** |
|  | FN1 | **0.00029** | **4.77** |
|  | COL3A1 | **0.00029** | **4.45** |
|  | COL1A2 | **0.00029** | **4.44** |
|  | COL11A1 | **0.00029** | **4.44** |
|  | COL5A2 | **0.00029** | **4.20** |
|  | COL5A1 | **0.00029** | **3.82** |
|  | THBS4 | **0.04767** | **2.64** |
| MuEp |  |  |  |
|  | COL1A1 | **0.00029** | **4.75** |
|  | COMP | **0.00581** | **4.51** |
|  | COL11A1 | **0.00029** | **3.72** |
|  | COL1A2 | **0.00029** | **3.46** |
|  | COL3A1 | **0.00042** | **3.32** |
|  | FN1 | **0.00042** | **3.25** |
|  | SPP1 | **0.00129** | **3.12** |
|  | COL5A1 | **0.00033** | **3.00** |
|  | COL5A2 | **0.00033** | **2.69** |
|  | LAMB3 | **0.00029** | **2.42** |
| AdCy |  |  |  |
|  | COMP | **0.00110** | **3.93** |
|  | COL27A1 | **0.00029** | **3.23** |
|  | COL1A1 | **0.03678** | **2.31** |
|  | COL5A1 | **0.00762** | **2.12** |
|  | LAMC2 | **0.00040** | **2.06** |
|  | THBS4 | 0.06272 | 1.83 |
|  | SPP1 | 0.15150 | 1.64 |
|  | COL2A1 | 0.10532 | 1.60 |
|  | COL5A2 | **0.04660** | 1.34 |
|  | COL11A2 | **0.01420** | 1.30 |

Table S2: Overview of *p*-values (tumour versus normal and interentity analysis) and log_2_-fold ratios (tumour versus normal) of all addressed genes.

Table S3: List of all COL27A1 peptides detected with MSI. Peptides were produced via in-silico digestion using the ProteinProspector MS-Digest tool (http://prospector.ucsf.edu). The corresponding MSI adduct masses are shown.
